# Refinement and Usability Analysis of an eHealth App for Ankylosing Spondylitis as a Complementary Treatment to Physical Therapy: Development and Usability Study

**DOI:** 10.2196/47426

**Published:** 2023-12-12

**Authors:** Marlies Nitschke, Obioma Bertrand Nwosu, Lara Grube, Johannes Knitza, Ann-Kristin Seifer, Bjoern M Eskofier, Georg Schett, Harriet Morf

**Affiliations:** 1 Machine Learning and Data Analytics Lab, Department Artificial Intelligence in Biomedical Engineering (AIBE) Friedrich-Alexander-Universität Erlangen-Nürnberg (FAU) Erlangen Germany; 2 Department of Internal Medicine 3- Rheumatology & Immunology Universitätsklinikum Erlangen Friedrich-Alexander-Universität Erlangen-Nürnberg Erlangen Germany; 3 Deutsches Zentrum Immuntherapie Universitätsklinikum Erlangen Friedrich-Alexander-Universität Erlangen-Nürnberg Erlangen Germany; 4 Translational Digital Health Group, Institute of AI for Health German Research Center for Environmental Health, Helmholtz Zentrum München Neuherberg Germany

**Keywords:** ankylosing spondylitis, axial spondylarthritis, DHA, digital health application, eHealth, self-assessment, Usability, Yoga, YogiTherapy

## Abstract

**Background:**

Mobile eHealth apps have been used as a complementary treatment to increase the quality of life of patients and provide new opportunities for the management of rheumatic diseases. Telemedicine, particularly in the areas of prevention, diagnostics, and therapy, has become an essential cornerstone in the care of patients with rheumatic diseases.

**Objective:**

This study aims to improve the design and technology of YogiTherapy and evaluate its usability and quality.

**Methods:**

We newly implemented the mobile eHealth app YogiTherapy with a modern design, the option to change language, and easy navigation to improve the app’s usability and quality for patients. After refinement, we evaluated the app by conducting a study with 16 patients with AS (4 female and 12 male; mean age 48.1, SD 16.8 y). We assessed the usability of YogiTherapy with a task performance test (TPT) with a think-aloud protocol and the quality with the German version of the Mobile App Rating Scale (MARS).

**Results:**

In the TPT, the participants had to solve 6 tasks that should be performed on the app. The overall task completion rate in the TPT was high (84/96, 88% completed tasks). Filtering for videos and navigating to perform an assessment test caused the largest issues during the TPT, while registering in the app and watching a yoga video were highly intuitive. Additionally, 12 (75%) of the 16 participants completed the German version of MARS. The quality of YogiTherapy was rated with an average MARS score of 3.79 (SD 0.51) from a maximum score of 5. Furthermore, results from the MARS questionnaire demonstrated a positive evaluation regarding functionality and aesthetics.

**Conclusions:**

The refined and tested YogiTherapy app showed promising results among most participants. In the future, the app could serve its function as a complementary treatment for patients with AS. For this purpose, surveys with a larger number of patients should still be conducted. As a substantial advancement, we made the app free and openly available on the iOS App and Google Play stores.

## Introduction

Ankylosing spondylitis (AS) is one of the most severe rheumatic diseases [1]. It affects the axial skeleton and, in some cases, the peripheral joints, entheses, skin, eyes, and gastrointestinal organs. Inflammation can especially affect the lower spine and cause stiffness, leading to immobility. These symptoms can interfere with daily activities and significantly impact people’s quality of life [2]. AS involves people of various ages. About 80% of the patients develop the first symptoms at an age younger than 30 years, but the disease duration is increasing [3]. Due to its early onset, severity, and high-level chronicity, AS is associated with a substantial burden on the individual and the health care system in Germany [4]. For this reason, it is important to start early treatment to avoid progressions such as chronic pain, bone damage, and ankylosis. Nowadays, a combination of medical treatments in the form of biologicals and regular exercises is efficient and safe to reduce inflammation and receive remission, which leads to a reduction of work disability and sick leaves (from 2000 to 2012, early retirement rates decreased from 19% to 14%) [4].

Besides drug treatments, physical therapy is important to improve well-being and prevent stiffness in patients with AS [5]. Yoga, a combination of stretching and strengthening exercises, has been accepted as a popular treatment for rheumatic and musculoskeletal diseases [6]. The combination of drugs and yoga has also shown better results than treatment with drugs only [7].

Over the years, there has been considerable growth in the use of mobile eHealth apps as supporting treatment for rheumatic diseases such as rheumatoid arthritis or systemic lupus erythematosus [8,9]. Particularly during the COVID-19 pandemic, mobile eHealth apps have been used as a complementary treatment to increase the well-being of patients [10]. Especially in the field of fitness apps and exercise therapy, there has been an increase in recent years [11]. Digital apps can be beneficial in preventing a bad course of the disease and could be a cost-efficient solution in helping health insurance companies save costs [12]. It also creates more time for patients, as exercises can be performed directly from home [13]. The aim is to save as much time as possible, as appointments with a physiotherapist are often difficult to get or specialized clinics are far away. Therefore, many patients look on the internet for ways to stay active. However, there is often a lack of specific programs adapted to the disease. From our experience, many patients also feel safe knowing that experts have put the exercises together, for example, when the exercise program is recommended by their physicians.

The problem with many fitness apps is the 1-sided benefit. Often only exercises can be performed, but the patient cannot assess the effect of the exercises on the course of the disease [12]. YogiTherapy tries to combine these 2 aspects. The mobile app was developed in 2020 to provide a training section with video instructions for yoga exercises specified for patients with AS and an assessment section giving the ability to monitor the progression of the disease [14]. In the assessment section, patients can conduct tests related to disease-specific mobility, function, disease activity, and quality of life. The results are then displayed graphically on a timeline to give the patient automatic feedback. The app also provides information with pictures on individual exercises, about the disease itself, and nutrition recommendations for rheumatic diseases.

The objective of this work was to improve the existing prototype of YogiTherapy and evaluate the app. In detail, we reimplemented YogiTherapy as a cross-platform app with the open-source tools React Native [15] and Expo [16] to create a modern design and intuitive navigation and to extend its functionalities. We included the option to change the language between English and German, with the possibility to add other languages easily. Furthermore, we added search and filtering functions to the training section and enabled exporting of the test results. With the refined app, we performed a study with patients with AS to evaluate the usability and quality. The study participants first performed a task performance test (TPT) with a think-aloud protocol and completed the German version of the Mobile App Rating Scale (MARS-G) afterward. Finally, we improved YogiTherapy based on the study and published it in the iOS App [17] and Google Play stores [18].

## Methods

### Features

Before reimplementing YogiTherapy, our team of computer scientists from the Machine Learning and Data Analytics Lab at the Friedrich-Alexander-Universität Erlangen-Nürnberg and rheumatologists at the University Hospital Erlangen analyzed our existing prototype and its previously identified usability issues [14]. Based on this, we define the following functional requirements and features:

#### Multilanguage Support

Compared to the previous version, we added multilanguage support so that the app supports English and German and can easily be expanded to other languages.

#### Welcome Page

**Figure 1:**
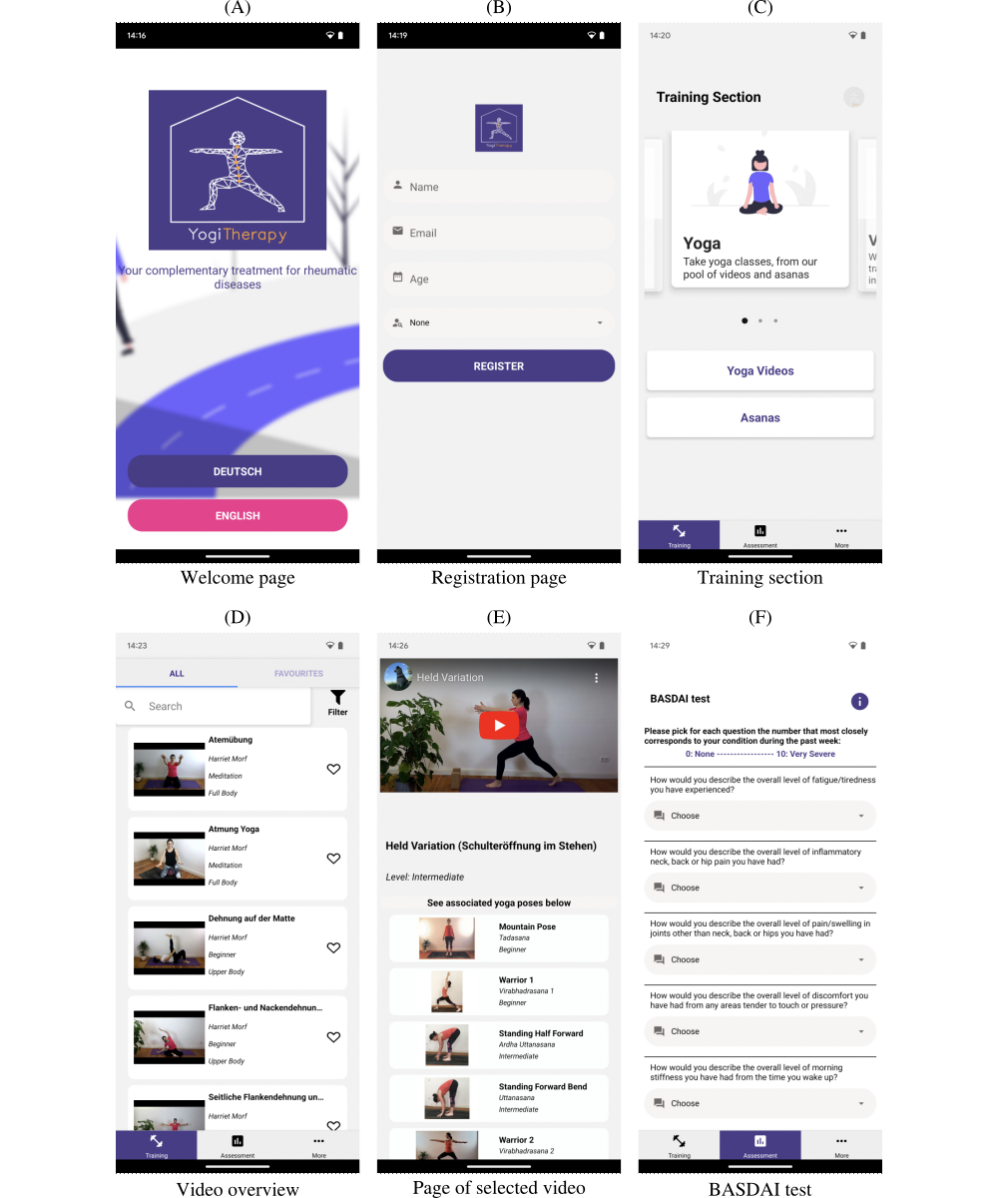
Screenshots of YogiTherapy after the refinement based on the study results. BASDAI: Bath Ankylosing Spondylitis Disease Activity Index.

The app shows a welcome page when using it for the first time to register and choose a language (Figure 1A and B).

#### Training Section

The app provides a yoga training section specifically developed for patients with AS by a rheumatologist and yoga teacher (Figure 1C-E). The patient can stream yoga exercise videos for independent training at home. Additionally, yoga poses (*asanas*) are explained to practice the correct execution. The initial app version additionally provided real-time feedback on the yoga pose execution using artificial intelligence [14]. However, as this functionality had high restrictions with respect to the smartphone’s hardware, we decided to remove it from this updated version to enable a broad user group. To increase usability, we recently introduced search, filter, and favorite options.

#### Assessment Section

In addition to the training section, the app allows the assessment of the training effect (Figure 1F). To this end, patients can complete 4 AS-specific tests (Ankylosing Spondylitis Quality of Life [ASQoL]; Bath AS Metrology Index [BASMI]; Bath AS Disease Activity Index [BASDAI]; and Bath AS Functional Index [BASFI]) [19]. Patients can monitor the test results over time as a graph to keep track of the disease progression. Additionally, we added the possibility of exporting the test progress to share it, for example, with the treating physician.

#### Information Page

The app summarizes nutritional advice and information about the disease on an information page to provide further insights.

### Software Development

We used the combination of React Native [15] and Expo [16], which are open-source tools for making universal native apps for Android, iOS, and the web with JavaScript. React Native was chosen because it provides a native look and fast reload. Our aim was an easy navigation experience for the user, so we used the inbuilt React Native navigation to design our navigation system to be achievable with the fewest clicks possible. We used the latest version of all libraries and frameworks available during app development. We realized multilanguage support for German and English using the library *reacti18next*. In the future, the app will be easily extendable to other languages. After the first start of the app, a welcome page is displayed to register with name, email address, age, and gender (Figure 1A). The information entered during registration was validated for plausibility using the libraries *formic* and *yup*. All user-specific information was stored only locally on the device, allowing the patients to maintain full control of their data.

The videos of the yoga exercises are streamed from YouTube within the YogiTherapy using the *react-native-youtube-iframe* library. In addition, we used the *react-native-community/netinfo* library to check for an internet connection to display the videos. To mark favorite videos and asanas, we used AsyncStorage, a persistent storage available in React Native. Furthermore, users can filter the videos and asanas according to fitness level and exercise type. The asanas associated with a yoga exercise are displayed below the video (Figure 1E).

In the assessment section, we provided AS-specific tests. The app stores a JavaScript object containing the test result, type, date, and unique ID in AsyncStorage. For sharing the disease progress, the test results can be exported as an Excel file using the library *Exceljs*.

### Participant Recruitment

The usability and quality evaluation of YogiTherapy was performed at the Medicine Clinic 3, Department of Rheumatology and Immunology at the University Hospital Erlangen, Germany. The study was carried out from May 2022 to September 2022. Patients were recruited by a medical doctoral student. Patients were approached in the rheumatology outpatient clinic waiting room to ask if they would like to participate in the survey. In addition, patients were also notified by letter, email, or telephone. The most common reasons for nonparticipation were a lack of time, no interest in surveys, no interest in yoga, or little experience with smartphones. The inclusion criteria were the diagnosis of AS according to the Assessment of SpondyloArthritis International Society classification criteria [20] and sufficient German language skills.

### Ethical Considerations

Participants were informed about the study and had to sign the informed consent form before participating. The study was approved by the ethical committee of the Friedrich-Alexander-Universität Erlangen-Nürnberg (application 8_21B). All collected data were pseudo-anonymized by assigning a participant ID, and video recordings were limited to the hands and smartphone screen. No monetary compensation was paid.

### Study Design

A total of 16 participants (4 female and 12 male) aged between 20 and 73 (mean 48.1, SD 16.8) years took part in the study. Patients had mild to moderate disease severity (mean BASMI score 0.79, SD 1.67). The majority were also affected by peripheral joint involvement (11/16, 69%). Furthermore, individual patients had ocular, skin (psoriasis), or intestinal involvement.

The 2 authors (a medical student and a computer science student) recruited the patients and conducted the main part of the interviews. Study participants had to perform the TPT with a think-aloud protocol and complete a questionnaire for the MARS-G. Upon arrival, we explained the protocol to the participants without showing them the app or questionnaire.

The TPT is a recognized and published test in web analytics and usability testing. The TPT included 5 tasks (Table 1; the German version is provided in Multimedia Appendix 1) that patients were asked to complete as quickly as possible using a provided phone (OnePlus Nord). The smartphone was connected to the internet. The data of previous participants were deleted from the app. The participants were provided with a printout of the tasks in German. To determine how easy it is to use the app and where difficulties can occur, we asked the participants to speak aloud while carrying out the tasks according to the think-aloud protocol. We used a GoPro (GoPro Inc) camera to record the smartphone screen and audio for subsequent analysis of the actions and thoughts of the participants.

**Table 1:** English translation of the tasks of the task performance test.

Number and name	Description
1. Create an account	Imagine that the app YogiTherapy has been recommended to you by your doctor, and you have now successfully downloaded it to your smartphone. Fill out the registration form with the following data and thus create your profile: Name: Test Email: demo@gmail.de Age: 33 Gender: None
2. Watch a yoga video	You have now successfully registered, and you are motivated to watch a video for your back. Now go to the yoga section of the app and start the video “Lying Backbend.”
3. Filter videos by properties	As you watch the video, you realize that the video is for advanced users. Now, to find videos for your beginner level, use the app’s filter function and search for beginner videos. How many are there?
4. Take a test	A small time shift; you have been using the app for a few days now and would like to see your improvement progress. To do this, find the test with the name BASDAI^a^. Click on the test and answer it completely. After that, please save the score.
5. Inspect your progress	You have now been doing your yoga exercises regularly and have also completed the BASDAI test several times. You are really proud of yourself for your discipline in doing the exercises. All you need is an overview of your progress. Now, look for this month’s progress in the BASDAI test in the app.
6. Share your progress	Wow, you are surprised about the great progress you have made. Share the progress of the BASDAI test with the doctor by email! The task is finished when you get to the email draft, so you do not need to fill in the email content.

^a^BASDAI: Bath Ankylosing Spondylitis Disease Activity Index.

After the TPT, the participants directly performed the MARS-G using an electronic questionnaire on a provided tablet [21,22]. In contrast to the first version of the app, which was evaluated with the user experience questionnaire [14], we chose the Mobile App Rating Scale (MARS) as it is among the most used questionnaires for assessing the quality of mobile eHealth apps [22]. The MARS is an assessment tool that helps evaluate a digital app [12,23]. The questionnaire contains the following sections: (A) engagement, (B) functionality, (C) aesthetics, (D) information quality, (E) subjective app quality, and (F) app specific. From section D (information quality), we removed the inapplicable items XIII, XIV, and XIX since no written app description was provided at the time of the study and patients were unaware of the previous app version. Every MARS item is answered on a 5-point scale (1=inadequate, 2=poor, 3=acceptable, 4=good, and 5=excellent).

### Evaluation

We viewed the video recordings of the TPT to determine if a task was successfully completed and how long it took to complete it [24]. We rated a task as successfully completed if the participant achieved the goal in not more than 120 seconds. The time started when the patients finished reading the instructions for the respective task. For evaluation, we determined the task completion rate per participant across all tasks by taking the number of completed tasks of this participant over the total number of tasks [25]. Similarly, we calculated the task completion rate per task across all participants and the overall task completion rate. Furthermore, we obtained the binomial 95% CIs for all completion rates using the adjusted Wald method [26]. To analyze how fast a task was completed, we averaged the completion time of each task across the participants and computed the respective 95% CIs using the Wald method. Finally, 1 assessor transcribed the participants’ verbal statements from the video recordings and clustered them without using predefined categories.

For each participant of the MARS-G, we computed the engagement, functionality, aesthetics, and information mean score by averaging the items of the respective sections. The average of those 4 sections is called the app quality mean score. Additionally, we computed the app’s subjective quality mean score for every participant by averaging the items of this section. The section F (app specific) serves only for descriptive purposes [23]. Therefore, the section result was not used as a score, in alignment with the original publication of the MARS [23]. Finally, we computed the mean and SD for all participants for each item and the mean scores.

## Results

**Figure 2:**
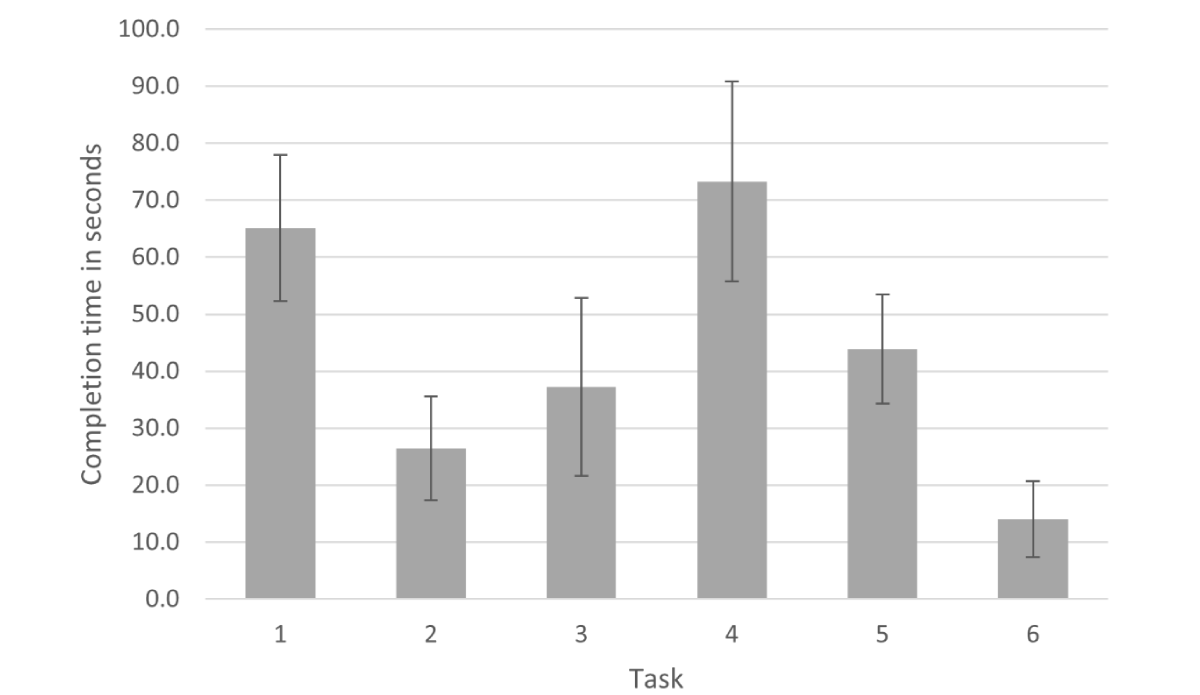
Average completion time per task for the task performance test over the 16 participants. Error bars present the 95% CI.

A total of 16 participants took part in the TPT, but 4 participants (IDs 3, 7, 10, and 13) did not complete the MARS questionnaire. Table 2 summarizes the task completions of the TPT. The overall completion (84/96, 88% completed tasks) was high. A total of 9 participants completed all tasks successfully; 4 participants failed only 1 of the 6 tasks, 1 participant failed 2 tasks, and 2 participants failed half (3/6, 50%) of the tests. Task 1 (create an account) and task 2 (watch a yoga video) had a completion rate of 100% (16/16). Task 1 (create an account) was easy to complete but required, on average, 65.1 seconds to complete, which was the second-highest completion time (Figure 2). Task 4 (take a test) had the lowest completion rate (11/16, 69%) and also the highest average completion time of 73.3 seconds. Task 6 (share your progress) needed, on average, the shortest time to be completed (14.1 s).

**Table 2:** Task completion of the task performance test. Successfully completed tasks are denoted by a check mark, and unsuccessful tasks by an empty cell. The completion rate is given per participant, per task, and overall. The binomial 95% CIs of the completion rates are given in parentheses.

Participant	Task 1	Task 2	Task 3	Task 4	Task 5	Task 6	Completion rate, % (95% CI)
1	✓	✓		✓	✓	✓	83.3 (41.8-98.9)
2	✓	✓		✓			50.0 (18.8-81.2)
3	✓	✓	✓		✓	✓	83.3 (41.8-98.9)
4	✓	✓	✓	✓	✓	✓	100 (55.7-100)
5	✓	✓	✓		✓	✓	83.3 (41.8-98.9)
6	✓	✓	✓	✓	✓	✓	100 (55.7-100)
7	✓	✓	✓	✓	✓	✓	100 (55.7-100)
8	✓	✓	✓		✓	✓	83.3 (41.8-98.9)
9	✓	✓	✓	✓	✓	✓	100 (55.7-100)
10	✓	✓	✓	✓	✓	✓	100 (55.7-100)
11	✓	✓	✓	✓	✓	✓	100 (55.7-100)
12	✓	✓	✓	✓	✓	✓	100 (55.7-100)
13	✓	✓				✓	50.0 (18.8-81.2)
14	✓	✓			✓	✓	66.7 (29.6-90.7)
15	✓	✓	✓	✓	✓	✓	100 (55.7-100)
16	✓	✓	✓	✓	✓	✓	100 (55.7-100)
Completion rate, % (95% CI)	100 (77.3-100)	100 (77.3-100)	75.0 (50.0-90.3)	68.8 (44.1-86.1)	87.5 (62.7-97.8)	93.8 (69.7-100)	87.5 (79.3-92.9)

In the think-aloud protocol, difficulties navigating the app became apparent. Especially, navigating back to a previous page could have been more intuitive for most participants, as they were unfamiliar with the double-click method. One user reclaimed the size of the text as it was too small to read. In addition, many participants could not find the filter icon since some entered the word “filter” into the search bar or did not know the filter icon. Furthermore, some participants had issues finding the assessment section, which we labeled with the word “Bewertung” in German. Even though all participants could start a yoga video (task 2), some had initial problems starting the video as they tried to click into the pictures of the associated asanas below the video.

For the MARS, the engagement, functionality, aesthetics, and information mean scores were good [21], resulting in a good app quality mean score of 3.79 (SD 0.51; Table 3). Compared to the app quality mean score, the app subjective quality score (3.52, SD 0.69) was slightly smaller, as two-thirds (8/12, 67%) of the participants were unsure whether they would pay for the app (item XXVI). Nevertheless, they would recommend the app to others (item XXIV) and use it frequently (item XXV). The evaluation also showed that YogiTherapy is interesting to use (item II); is target specific (item V); and has good performance (item VI), layout (item X), and graphics (item XI). However, customization possibilities (item III) could be improved.

**Table 3:** Frequency of how often the individual scale from 1 to 5 was selected and mean and SD of the German version of the Mobile App Rating Scale of all 12 participants.

Subscale and items		Frequency of scale (n=12), n (%)						Score, mean (SD)
		1	2	3	4	5		
**(A) Engagement mean score**								3.60 (0.28)
	I. Entertainment	0 (0)	0 (0)	6 (50)	5 (42)	1 (8)	3.58 (0.64)	
	II. Interest	0 (0)	0 (0)	3 (25)	3 (25)	6 (50)	4.25 (0.83)	
	III. Customization	2 (17)	0 (0)	7 (58)	3 (25)	0 (0)	2.92 (0.95)	
	IV. Interactivity	0 (0)	0 (0)	8 (67)	4 (33)	0 (0)	3.33 (0.47)	
	V. Target group	0 (0)	0 (0)	2 (17)	9 (75)	1 (8)	3.92 (0.49)	
**(B) Functionality mean score**								3.92 (0.55)
	VI. Performance	0 (0)	0 (0)	1 (8)	6 (50)	5 (42)	4.33 (0.62)	
	VII. Ease of use	1 (8)	0 (0)	3 (25)	7 (58)	1 (8)	3.58 (0.95)	
	VIII. Navigation	0 (0)	0 (0)	3 (25)	6 (50)	3 (25)	4.00 (0.71)	
	IX. Gestural design	0 (0)	0 (0)	6 (50)	3 (25)	3 (25)	3.75 (0.83)	
**(C) Aesthetics mean score**								4.00 (0.72)
	X. Layout	0 (0)	0 (0)	4 (33)	4 (33)	4 (33)	4.00 (0.82)	
	XI. Graphics	0 (0)	0 (0)	3 (25)	5 (42)	4 (33)	4.08 (0.76)	
	XII. Visual appeal	0 (0)	0 (0)	4 (33)	5 (42)	3 (25)	3.92 (0.76)	
**(D) Information mean score**								3.63 (0.67)
	XV. Quality of information	0 (0)	0 (0)	3 (25)	7 (58)	2 (17)	3.92 (0.64)	
	XVI. Quantity of information	0 (0)	0 (0)	5 (42)	6 (50)	1 (8)	3.67 (0.62)	
	XVII. Visual information	0 (0)	0 (0)	4 (33)	6 (50)	2 (17)	3.83 (0.69)	
	XVIII. Credibility	3 (25)	1 (8)	2 (17)	4 (33)	2 (17)	3.08 (1.44)	
App quality mean score								3.79 (0.51)
**(E) App subjective quality score**								3.52 (0.69)
	XXIV. Would you recommend this app?	0 (0)	1 (8)	3 (25)	4 (33)	4 (33)	3.92 (0.95)	
	XXV. How many times do you think you would use this app?	0 (0)	1 (8)	2 (17)	6 (50)	3 (25)	3.92 (0.86)	
	XXVI. Would you pay for this app?	3 (25)	0 (0)	8 (67)	0 (0)	1 (8)	2.67 (1.11)	
	XXVII. What is your overall star rating of the app?	0 (0)	0 (0)	5 (42)	7 (58)	0 (0)	3.58 (0.49)	

## Discussion

This study aimed to improve YogiTherapy as a mobile eHealth app that can serve as an additional non–drug-based treatment for patients with AS. YogiTherapy provides yoga videos tailored to patients with AS and allows tracking of disease progression with standardized assessment tests. Overall, the app’s quality was good, which was demonstrated by a high task completion rate in the TPT and a high score in the MARS questionnaire.

In the TPT, the participants could complete most tasks without any previous explanation, resulting in an overall performance rate of 88% (84/96 completed tasks). Hence, the app use can be considered intuitive. Participants with the largest difficulties who could not complete 2 or more tasks were 60 years of age or older. Therefore, the differences depending on age might indicate that YogiTherapy might be harder to operate for less experienced users. However, we did not record how experienced the participants were with using smartphones and apps. This should be taken into consideration in follow-up studies.

To further improve the usability of YogiTherapy based on the study results, we analyzed task 3 (filter videos by properties) and task 4 (take a test) in more detail since they had the lowest success rates. The think-aloud protocol revealed that even participants who completed all tasks successfully had difficulties recognizing or finding the filter icon in task 3. Hence, we increased the size of the filter icon and added the label “Filter” as an explanation below the icon (Figure 1D). Furthermore, participants had issues finding the tests in task 4 since they did not associate the German translation of “assessment” (*Bewertung*) with the tests (Figure 1C). Based on this feedback, we renamed the section in the German translation “Test/Fortschritt.” This case demonstrates the importance of proper translations and correct wording.

The TPT was also performed on a previous version of the YogiTherapy app [14], but we used new tasks to also cover the added functionalities of the revised version and did not allow the participants to familiarize themselves with the app before the test. Nevertheless, 3 of the tasks were comparable between the studies. In both studies, all participants could successfully watch a yoga exercise video (task 1 in the previous study and task 2 in this study). However, in the previous version, the participants could start an arbitrary video while they had to search and start a specific video in this study. Hence, the level of difficulty of this task was higher in this study. The BASDAI test could be completed by 90% of the participants in the previous study, which was conducted in English, and only by 69% (11/16) of this study, which was conducted in German (task 5 in the previous study and task 4 in this study). The decrease in the completion rate of this task was most probably caused by the misleading German translation of “assessment,” as discussed earlier. In the previous study, 80% of the participants could visualize the results of the BASMI test (task 4). After the refinement of the app, the task completion rate slightly increased, as 88% (14/16) of the participants could visualize the results of the BASDAI test in this study (task 5).

The results from the MARS showed that every section scored above average from the participants’ point of view. YogiTherapy had an app quality mean score of 3.79 (SD 0.51) and thus exceeded the average score of 3.17 (SD 0.75) of 18 pain-related mobile apps [27] and the average score of 3.48 (SD 0.28) of 60 chronic condition or multimorbidity apps [28]. This implies that the app is readily acceptable and can be used by intended patients with AS [29].

The graphics item XI had one of the highest scores (4.08, SD 0.76), which can be attributed to the well-thought-out graphics used in developing YogiTherapy. We used representative images, and the layout was consistent and straightforward. The functionality mean score (3.92, SD 0.55) was the second-highest section score of the MARS. The good results in the navigation item VIII contradict the findings of the TPT, where several participants had issues navigating the app. However, this contradiction can be clarified because the study conductor explained to the participants how to navigate after the TPT. While this is a restriction of the presented study, it highlights that participants understood how the navigation works after explanation. We built the design so that the previous screen could be accessed with a finger wipe or a double-click, so the participants thought it was efficient and adequate. After the study, we added an explanation of the navigation to the welcome page to introduce this functionality. The performance item VI obtained the highest item score as we focused on the performance during implementation. For example, we bundled the pictures of the asanas into the app. Except for functionalities like watching yoga videos and sharing results, all functionalities work without an internet connection, providing less latency delay and a more pleasant experience for users.

The engagement section of the MARS gives important insights since YogiTherapy aims to be a target-specific app for patients with AS. Most of the study participants agreed that the YogiTherapy is target-specific, as the target item V reached a score of 3.92 (SD 0.49). On the other side, more customization features, such as sound or content notifications, could be added to YogiTherapy. The participants also indicated that the interactivity (item IV) of YogiTherapy is just about average. However, the participants are highly interested in the project and are very willing to use YogiTherapy as a complementary solution to their challenge.

Compared to other fitness apps (eg, Daily Yoga [30]), YogiTherapy was developed specifically for patients with AS by doctors, computer scientists, and medical professionals. Nevertheless, the participants doubted the credibility (item XVIII) of the information provided in the app. The low score could originate from most participants not seeing the well-referenced information section before participating in the MARS. Credibility could be increased by the treating physician recommending YogiTherapy.

The MARS of our app was in the middle of 9 mobile apps for patients with rheumatic diseases, for which the app quality mean scores ranged from 3.44 to 4.19 [12]. The app Rheuma Auszeit was developed with a patient organization and reached the highest MARS. Rheuma Auszeit was the only 1 of the 9 apps providing instructions for mental and physical exercises. However, it did not provide a functionality to track the disease progression [12]. Like our app, Rheuma Auszeit does not allow customization, which also led to a low score for this item (Rheuma Auszeit: 2.5; YogiTherapy: 2.9) [31]. The other 8 apps featured progression tracking, but only 3 of them used validated questionnaires, which are established in clinical practice [12]. In contrast to those apps, YogiTherapy fully accompanies patients by giving exercise instructions and allowing the disease status to be assessed with validated tests. Nevertheless, YogiTherapy could further benefit from customization functionality and patient input.

Despite the promising results, the presented usability study had several limitations. The most important limitation lies in the small study population of 16 participants for the TPT and 12 for the MARS recruited in a single hospital due to time restrictions. In addition to patients, who are the end users, domain experts, health care professionals, and physiotherapists should be included in the study population. Furthermore, participant’s typical physical activity and experience with digital technologies, especially apps in the fitness sector, should be assessed. Even though the MARS is widely established, the mHealth App Usability Questionnaire (MAUQ) [32] could be a valuable alternative, as it was designed to determine the usability of mobile health apps [22].

In conclusion, the refined YogiTherapy app exhibited promising outcomes in usability testing among most participants. In the future, additional research should also focus on how patients use YogiTherapy daily and evaluate whether the app can motivate them to be physically more active. Moreover, there remain small improvement possibilities in the implementation to further enhance the app’s intuitiveness. Overall, we demonstrated that YogiTherapy is a user-friendly app specified for patients with AS, providing yoga videos for physical exercise and disease-specific tests to monitor disease progression. We made YogiTherapy accessible in the iOS App [17] and Google Play stores [18] to make it widely available to patients.

## Appendix

Multimedia Appendix 1 German version of the task performance test.

## Abbreviations

AS: Ankylosing Spondylitis
ASQoL: Ankylosing Spondylitis Quality of Life
BASDAI: Bath Ankylosing Spondylitis Disease Activity Index
BASFI: Bath Ankylosing Spondylitis Functional Index
BASMI: Bath Ankylosing Spondylitis Metrology Index
MARS: Mobile App Rating Scale
MARS-G: German version of the Mobile App Rating Scale
MAUQ: mHealth App Usability Questionnaire
TPT: Task Performance Test

## Data Availability

The data sets generated and analyzed during this study are available from the corresponding author upon reasonable request. The majority of the data are included in this published article.
