# Supplementary material for: Refinement and Usability Analysis of an eHealth App for Ankylosing Spondylitis as a Complementary Treatment to Physical Therapy: Development and Usability Study
Source: JMIR Form Res. 2023 Dec 12;7:e47426. doi: 10.2196/47426 (PMC10751630; doi:10.2196/47426)
Supplement: Multimedia Appendix 1 [file formative_v7i1e47426_app1.docx]

**Appendix Table 1.** Original German version of the tasks of the task performance test.

| Number and name | Description |
| --- | --- |
| 1. Einen Account erstellen | Stellen Sie sich vor, Ihnen wurde von Ihrem Arzt die App YogiTherapy empfohlen, und Sie haben diese nun erfolgreich auf ihr Handy geladen. Füllen Sie die Registrierung mit den folgenden Daten aus und erstellen Sie somit Ihr Profil:  • Name: Test  • E-mail: demo@gmail.de  • Alter: 33  • Geschlecht: Kein |
| 1. Ein Yoga Video anschauen | Sie haben sich nun erfolgreich registriert und sind motiviert ein Video für Ihren Rücken anzuschauen. Gehen Sie nun in den Yoga-Bereich der App und starten Sie das Video „Rückbeuge im Liegen“. |
| 1. Videos nach Eigenschaften filtern | Beim Anschauen merken Sie, dass das Video doch für fortgeschrittenere Nutzer geeignet ist. Um nun Videos für Ihr Anfängerniveau zu finden, nutzen Sie die Filterfunktion der App und suchen nach Anfänger-Videos. Wie viele gibt es da? |
| 1. Einen Test absolvieren | Ein kleiner Zeitsprung, Sie nutzen nun die App bereits seit einigen Tagen und möchten gerne Ihren erbesserungsfortschritt sehen. Suchen Sie dafür den Test mit dem Namen BASDAI. Klicken Sie auf den Test und beantworten ihn vollständig. Danach speichern Sie bitte die Punktzahl. |
| 1. Fortschritt sehen | Sie haben nun regelmäßig Ihre Yogaübungen durchgeführt und auch den BASDAI Test mehrmals ausgefüllt. Sie sind wirklich stolz auf sich, wie diszipliniert Sie die Übungen gemacht haben. Jetzt fehlt Ihnen nur noch der Überblick über Ihren Fortschritt. Suchen Sie nun in der App den Progress im BASDAI Test von diesem Monat. |
| 1. Fortschritt teilen | Wow, Sie sind überrascht über den großen Fortschritt, den Sie gemacht haben. Teilen Sie den Fortschritt im BASDAI Test per Email mit dem Arzt! Wenn Sie zu dem Email-Entwurf gelangt sind, ist die Aufgabe beendet, sodass Sie den Email-Inhalt nicht auszufüllen brauchen. |

^a^BASDAI: Bath Ankylosing Spondylitis Disease Activity Index.
